# Engagement With Gamification Elements in a Smoking Cessation App and Short-term Smoking Abstinence: Quantitative Assessment

**DOI:** 10.2196/39975

**Published:** 2023-02-01

**Authors:** Nikita B Rajani, Luz Bustamante, Dominik Weth, Lucia Romo, Nikolaos Mastellos, Filippos T Filippidis

**Affiliations:** 1 Department of Primary Care and Public Health Imperial College London London United Kingdom; 2 NextStage Consulting Dubai United Arab Emirates; 3 Laboratoire EA 4430-Clinique Psychanalyse Developpement Department of Psychology University of Paris Nanterre Paris France; 4 Research and Development Department Kwit SAS Strasbourg France; 5 Inserm–Le Centre de Recherche en Epidémiologie et Santé des Populations 1018 UPS Hôpital Raymond-Poincaré Paris France

**Keywords:** gamification, smoking cessation, smoking abstinence, mHealth, mobile apps, mobile phone, smartphone, digital health, user engagement, cognitive outcome, self-support, in-app metrics

## Abstract

**Background:**

Gamification in smoking cessation apps has been found to improve cognitive outcomes associated with higher odds of quitting. Although some research has shown that gamification can also positively impact behavioral outcomes such as smoking cessation, studies have largely focused on physical activity and mental health. Only a few studies have explored the effects of gamification on smoking cessation outcomes, of which the majority have adopted qualitative methodologies and/or assessed engagement with apps using self-report.

**Objective:**

This study aimed to explore levels of user engagement with gamification features in a smoking cessation app via in-app metrics. Specifically, the objective of this paper was to investigate whether higher engagement with gamification features is associated with the likelihood of quitting in the short term.

**Methods:**

Data from a larger online study that recruited smokers seeking to quit were analyzed to address the objectives presented in this paper. The study took place between June 2019 and July 2020, and participants were primarily recruited via social media posts. Participants who met the eligibility criteria used 1 of 2 mobile apps for smoking cessation. In-app metrics shared by the developer of one of the smoking cessation apps, called Kwit, were used to assess engagement with gamification features. Out of 58 participants who used the Kwit app, 14 were excluded due to missing data or low engagement with the app (ie, not opening the app once a week). For the remaining 44 participants, mean (SD) values were calculated for engagement with the app using in-app metrics. A logistic regression model was used to investigate the association between engagement with gamification and 7-day smoking abstinence.

**Results:**

In total, data from 44 participants who used the Kwit app were analyzed. The majority of participants were male, married, and employed. Almost 30% (n=13) of participants self-reported successful 7-day abstinence at the end of the study. On average, the Kwit app was opened almost 31 (SD 39) times during the 4-week study period, with the diary feature used the most often (mean 22.8, SD 49.3). Moreover, it was found that each additional level unlocked was associated with approximately 22% higher odds of achieving 7-day abstinence after controlling for other factors such as age and gender (odds ratio 1.22, 95% CI 1.01-1.47).

**Conclusions:**

This study highlights the likely positive effects of certain gamification elements such as levels and achievements on short-term smoking abstinence. Although more robust research with a larger sample size is needed, this research highlights the important role that gamification features integrated into mobile apps can play in facilitating and supporting health behavior change.

## Introduction

Despite steps taken to tackle the global tobacco epidemic, smoking-related health disability and mortality remain concerning [1]. While the majority of smokers want to quit smoking, research shows that in many countries such as the United Kingdom, long-term abstinence rates remain low [2]. In addition to low smoking cessation rates, face-to-face access to cessation services has been falling in several countries [3]. Digital health solutions, such as mobile apps, have been found to be effective methods of reaching individuals unwilling or unable to access in-person services. However, low engagement and retention are common challenges for mobile apps. The integration of gamification, the use of game elements in a nongame context [4], has been found to be positively associated with higher app engagement [5,6]. Some examples of gamification elements, also known as on-screen features or tactics, include goal setting, levels and badges, progress tracking, and progress sharing. Since gamification shares key elements with behavior change theories, it is often applied to health behavior change interventions [6].

Prior research shows that engagement with gamification elements in smoking cessation apps can positively impact cognitive constructs vital for abstinence such as self-efficacy and motivation to quit [7]. Although research also shows the positive effects of gamification on behavioral outcomes, the majority of existing studies have focused on physical activity and mental health [8]. A few studies that explored the association between engagement with gamification and smoking cessation adopted qualitative methodologies and assessed engagement using self-reported data rather than objective app usage metrics [6,7]. We aimed to explore the level of user engagement with gamification elements in a smoking cessation app via the analysis of in-app metrics. We also aimed to investigate whether engagement with gamification elements is associated with the likelihood of achieving short-term smoking abstinence.

## Methods

### Study Overview

The data used for this paper’s analyses were collected as part of a larger online study that took place from June 2019 to July 2020 and explored the effects of gamification on cognitive constructs vital for smoking cessation [7]. Participants were recruited via posters in public places in London, United Kingdom, and posts on various social media channels such as Facebook and Instagram. Interested participants were asked to fill out a screening questionnaire to assess eligibility. Eligible participants (Table S1, Multimedia Appendix 1) were assigned 1 of 2 smoking cessation apps; this paper focuses on users of Kwit, a gamified mobile app designed to help smokers quit smoking using cognitive behavior therapy [9]. Participants had to use the app at least once a week for 4 weeks and complete a questionnaire before (baseline), 2 weeks after (mid-study), and 4 weeks after app use (end of study). In-app metrics shared by the app developer were used to assess engagement with features. Out of the 70 participants who were assigned Kwit, 58 (83%) completed the study. A participant was considered to have completed the study if they self-reported to have engaged with the app at least once a week for the duration of the study and completed all questionnaires. Aside from free app access, participants had a chance to win a £50 (US $60) Amazon voucher.

### The Kwit App

**Figure 1:**
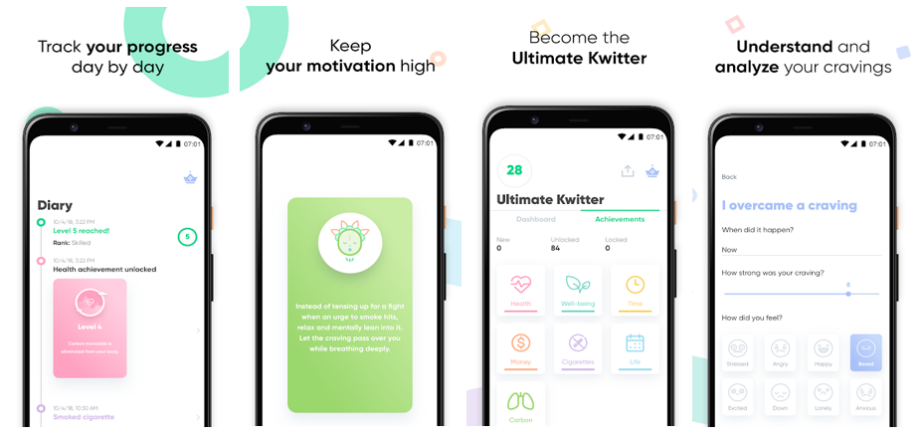
Screenshots of the Kwit app.

Kwit is a mobile app, developed by Kwit SAS, that aims to help smokers quit and successfully remain abstinent from smoking. It uses cognitive behavior therapy principles and gamification to assist individuals [9]. The app consists of features such as a smoking diary to log cravings and triggers, motivation cards, and a calculator/tracker to monitor self-progress in relation to key achievements. Figure 1 presents screenshots of the app, showing the smoking diary feature, an example of a motivation card, the achievements tracking page, and how users can track cravings. App versions 4.1 to 4.4 (June 2019 to July 2020) were used by participants.

### Measures

Sociodemographic measures included age (18-29 years, 30-41 years, 42-53 years, and 54-65 years), gender (male or female), marital status (single, married, or civil partnered), and employment status (unemployed: individuals willing or able to work but not employed; employed; nonemployed: students, individuals unable to work, and homemakers). Similar to other studies, the Fagerström questionnaire was used to assess nicotine dependence with responses categorized as low (0-4 points), moderate (5-7 points), and high (8-10 points) [10,11]. Additionally, to measure abstinence, participants were asked at the end of the study, “Have you smoked at all in the past seven days?” to assess the 7-day point prevalence of smoking abstinence. Participants who responded “No, not even a puff” were considered short-term quitters. Participants who selected other responses (“Yes, just a few puffs”; “Yes, between 1 and 5 cigarettes”; and “Yes, more than 5 cigarettes”) were categorized as smokers.

Aside from self-reported data, objective in-app metrics were also used in the analysis. Kwit SAS routinely collects and maintains a database of app usage statistics, including various user interactions with specific features and the app overall. Kwit SAS provided in-app metrics for the participant identification numbers shared with them. The metrics included the number of times the app was opened, levels completed, smoking diaries logged, achievements unlocked, and motivation cards viewed. Additional information on the in-app metrics is presented in Table S2 (Multimedia Appendix 1).

### Statistical Analysis

The statistical software Stata 16 (StataCorp) was used for the analysis. Descriptive statistics were used to present general participant characteristics, nicotine dependence, and 7-day abstinence. Mean (SD) values were calculated for the in-app metrics. Unadjusted logistic regression models were run for each in-app metric to explore its association with 7-day abstinence. The specifications of the adjusted logistic regression model were based on an iterative process that considered collinearity. Significance was set at the 5% level (*P*<.05), and 95% CIs were presented.

### Ethics Approval

The study was conducted in accordance with the recommendations for physicians involved in research on human subjects adopted by the 18th World Medical Assembly Declaration of Helsinki 1964 and later versions.

Ethical approval was obtained from the Joint Research Imperial College London Research Ethics Committee prior to the beginning of the study (reference: 19IC5158).

## Results

Among the 58 participants who completed the study (self-reported app usage once a week over the study duration and completion of all questionnaires), 14 participants were excluded from the analysis due to issues with the in-app metrics (eg, missing or inconsistent data; n=9) or due to inadequate engagement according to the in-app data (ie, the app was not used once a week; n=5). Table 1 shows that more than half of Kwit app users were 18 to 29 years of age (n=24, 55%). A majority were male (n=27, 61%), married (n=35, 80%), and employed (n=28, 64%). Moreover, the majority of participants had low to moderate dependence on nicotine (n=42, 95%). Almost a third (n=13, 30%) reported at the end of the study that they successfully abstained from smoking in the past 7 days.

As seen in Table 2, Kwit users opened the app almost 31 (SD 39) times on average over the 4-week study period. Among the metrics that were collected, the most frequently used features were logging of smoking diaries (mean 22.8, SD 49.3) and unlocking of achievements (mean 22.3, SD 16.5). Additionally, over the study period, motivation cards were opened on average 8.0 (SD 11.2) times and 7.7 (SD 4.9) levels were unlocked by Kwit users.

In the adjusted logistic regression model (Table 3), each additional level unlocked was associated with approximately 22% higher odds of achieving 7-day abstinence after controlling for other factors such as age and gender (odds ratio 1.22, 95% CI 1.01-1.47). The number of diaries logged and motivation cards opened were not significantly associated with 7-day abstinence in the adjusted model. The number of achievements was not included in the model since it was highly correlated with the number of levels. However, when the logistic regression model was rerun with the number of achievements instead of levels, the results suggested that each interaction with the achievements feature was associated with a 7% increased likelihood of reporting 7-day abstinence (95% CI 1.01-1.15).

**Table 1:** Characteristics of the study sample.

Characteristics		Participants (N=44), n (%)
**Age**		
	18-29 years	24 (55)
	30-41 years	13 (30)
	42-53 years	4 (9)
	54-65 years	3 (7)
**Gender**		
	Male	27 (61)
	Female	17 (39)
**Marital status**		
	Single	9 (21)
	Married or civil partnered	35 (80)
**Employment status**		
	Employed	28 (64)
	Nonemployed	13 (30)
	Unemployed	2 (5)
	Prefer not to answer	1 (2)
**Nicotine dependence**		
	Low (0-4 points)	25 (57)
	Moderate (5-7 points)	17 (39)
	High (8-10 points)	2 (5)
**7-day smoking abstinence**		
	Yes	13 (30)
	No	31 (70)

**Table 2:** Summary of engagement with mobile app features after 4 weeks of app use.

In-app metric	Value, mean (SD)
Number of times the app was opened	30.8 (39)
Number of motivation cards opened	8 (11.2)
Number of achievements unlocked	22.3 (16.5)
Number of diaries logged	22.8 (49.3)
Number of levels completed	7.7 (4.9)

**Table 3:** Logistic regression investigating the association between gamification and 7-day smoking abstinence at the end of the study (N=44).

Variable		7-day smoking abstinence, OR^a,b^ (95% CI)
Age		0.98 (0.91-1.05)
**Gender**		
	Male	Ref^c^
	Female	2.27 (0.45-11.52)
Number of diaries logged		0.97 (0.91-1.03)
Number of motivation cards opened		1.03 (0.97-1.11)
Number of levels unlocked		1.22 (1.01-1.47)

^a^OR: odds ratio.

^b^Adjusted for all the variables included in the table.

^c^Ref: referent.

## Discussion

### Principal Findings

We found that after 4 weeks of app use, almost 30% of smokers reported 7-day abstinence. This rate is generally within the range reported by other mobile app studies [12]. However, direct comparisons are difficult due to differences between interventions and varying methods of measuring cessation. Mobile app interventions such as Kwit can be associated with an increased likelihood of abstinence compared to no assistance or using willpower alone [13]. This study opens the possibility of using smoking cessation apps to aid individuals who are not accessing face-to-face services [14].

The analysis also found that engaging with levels was significantly associated with 7-day abstinence. According to Gnauk et al [15], levels are important as they can function as a goal-setting tool that marks progression and signals accomplishment. Similarly, the achievements or badges feature was also found to be associated with an increased likelihood of reporting abstinence. Achievements and levels are similar as they both provide regular feedback to users and remind them of their successes. This can lead to an increase in perceived competence, which facilitates health behavior change [16]. Generally, the positive effect of providing regular feedback in both remote and face-to-face interventions is well established [17]. Aside from the importance of features such as levels and achievements, it was also found that the likelihood of reporting abstinence was not statistically different with regards to age and gender after adjusting for engagement with app features. This could imply that any effects that Kwit may have on smokers might not vary by age or gender. However, it is important to note that the small sample size could impact the generalizability of the findings.

### Limitations

The observational nature of the study does not allow for causal inference; future research could carry out a rigorously designed randomized controlled trial with apps that differ only with regards to the type and number of gamification features. Aside from the sample size, the attrition of participants from 70 to 44 participants could pose a threat to the internal validity of the study and limit the generalizability of the findings. Additionally, since research has shown that relapses in abstinence can occur over a longer period of time, the relationship between engagement with gamification features and long-term quitting cannot be determined; future research could consider having a longer follow-up period [18,19]. Furthermore, validating smoking abstinence using biochemical verification is the gold standard and would be recommended to increase the robustness of future research studies.

Although the use of in-app metrics in mobile app studies is sparse and provides an objective method of assessing engagement with app features, it may not have captured the full engagement experience of users. For example, we were able to assess the number of times users engaged with specific gamification elements but not the length of engagement. While this is an imperfect measure of engagement, it can be considered a more objective method (compared to self-report) that is not frequently adopted in mobile app studies.

### Conclusions

The overall learnings from this research highlight that features such as levels and achievements can positively impact short-term smoking abstinence. While further investigation is warranted with a larger, diverse sample and a longer follow-up period, our findings have positive implications for the use of gamification in mobile apps to support behavioral outcomes such as smoking cessation.

## Appendix

Multimedia Appendix 1 Eligibility criteria and in-app metrics.
