# Supplementary material for: Engagement With Gamification Elements in a Smoking Cessation App and Short-term Smoking Abstinence: Quantitative Assessment
Source: JMIR Serious Games. 2023 Feb 1;11:e39975. doi: 10.2196/39975 (PMC9932870; doi:10.2196/39975)
Supplement: Multimedia Appendix 1 [file games_v11i1e39975_app1.docx]

**Multimedia Appendix 1. Eligibility criteria and in-app metrics.**

Table S1. Eligibility Criteria

| **Eligibility Criteria** |
| --- |
| 18 + years old |
| Smoked at least 100 cigarettes in lifetime and currently smokes at least one cigarette a day |
| Trying or willing to quit smoking in the next 30 days |
| Not using other forms of cessation treatment (e.g., NRT, medications, e-cigarettes) |
| Has not previously used the mobile application Kwit or Quit Genius and is not currently using any mobile app for smoking cessation |
| Has an Apple iPhone (5th generation or higher) or Android phone (version 18 or higher) |
| Not diagnosed with a mental health condition |

Table S2. In-App Metrics

| **Description/Feature** | **In-App Metric** |
| --- | --- |
| Overall engagement with the app | Number of times app was opened |
| Levels: 28 levels where the user can advance to the next level by achieving certain milestones (e.g., not smoking for a certain number of days). | Number of steps/levels completed |
| Smoking diaries: These allow users to log daily smoking statuses, cravings (and their intensity level) and triggers. | Number of diaries logged |
| Motivation Cards: These could “opened” by app users and contained messages to motivate the user to continue their quit journey. | Number of motivation cards opened |
| Achievements: There are 72 achievements in the Kwit app which a participant can unlock; each achievement is associated with a specific category (e.g., health, well-being, money, time, cigarettes and carbon dioxide levels). | Number of achievements unlocked |
